# Supplementary material for: Differential Gene and Protein Expressions Responsible for Vasomotor Signaling Provide Mechanistic Bases for the Opposite Flow-Induced Responses of Pre- and Post-Circle of Willis Arteries
Source: Life (Basel). 2025 May 26;15(6):856. doi: 10.3390/life15060856 (PMC12194257; doi:10.3390/life15060856)

Expression of COX1/2 and TXA2R proteins in rat middle cerebral (MCA) and basilar (BA) arteries

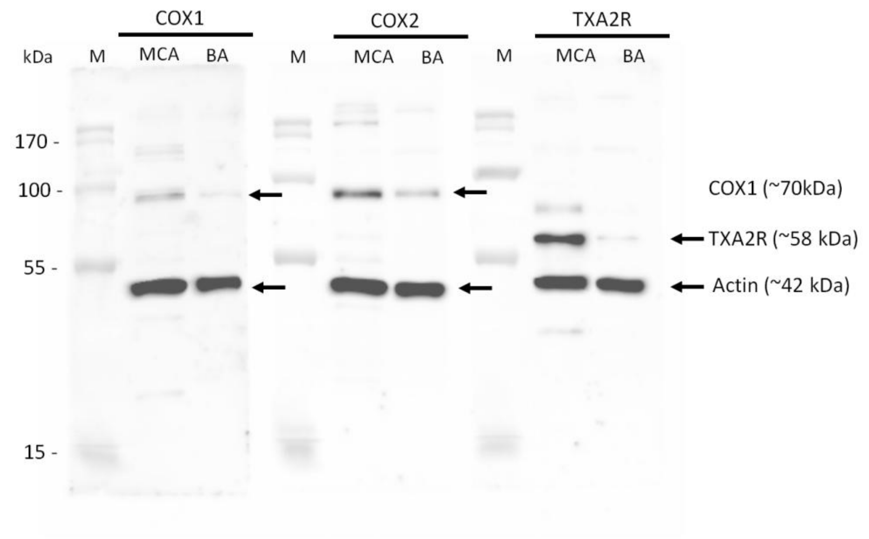

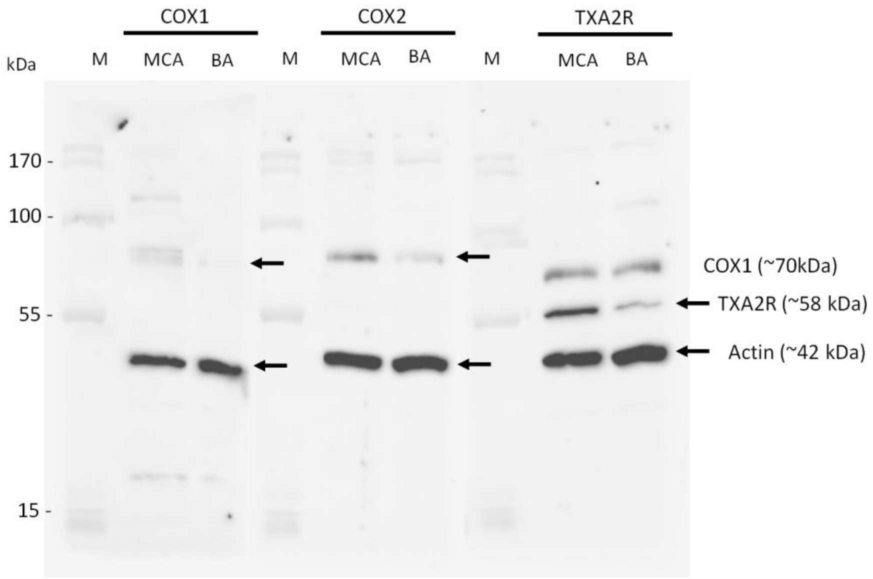

Expression of COX1/2 and TXA2R proteins in rat middle cerebral (MCA) and basilar (BA) arteries

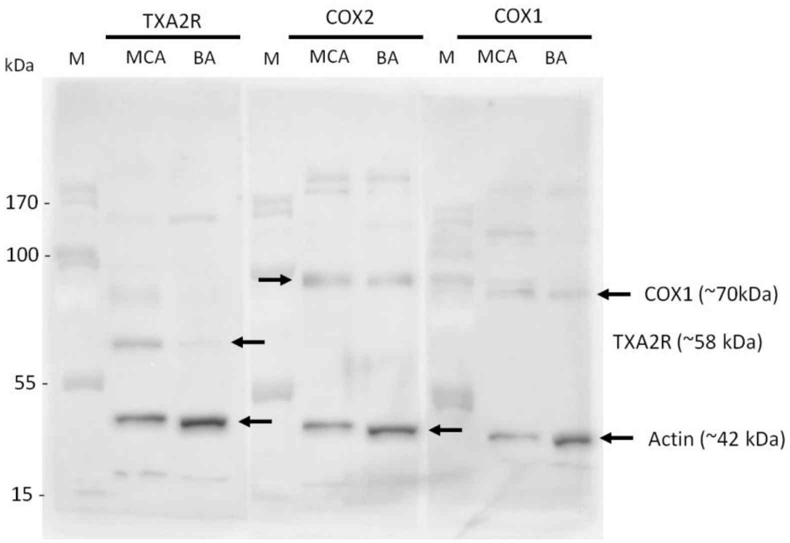

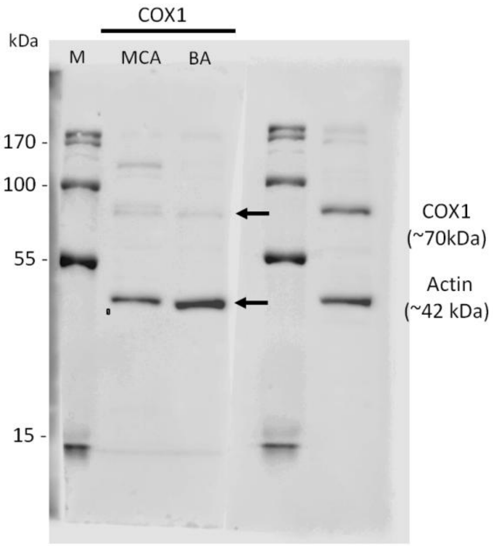

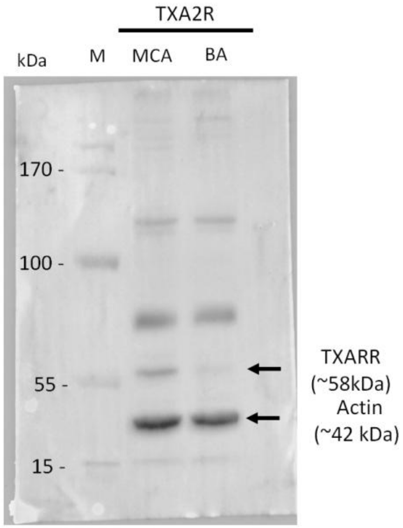

Expression of TXAS protein in rat middle cerebral (MCA) and basilar (BA) arteries

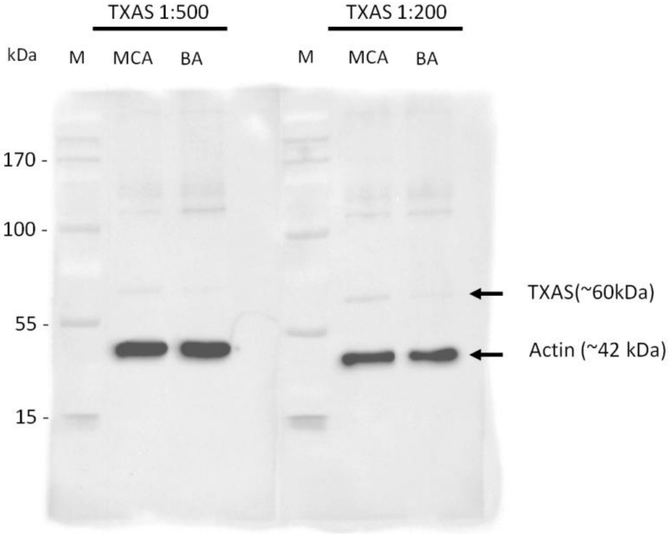

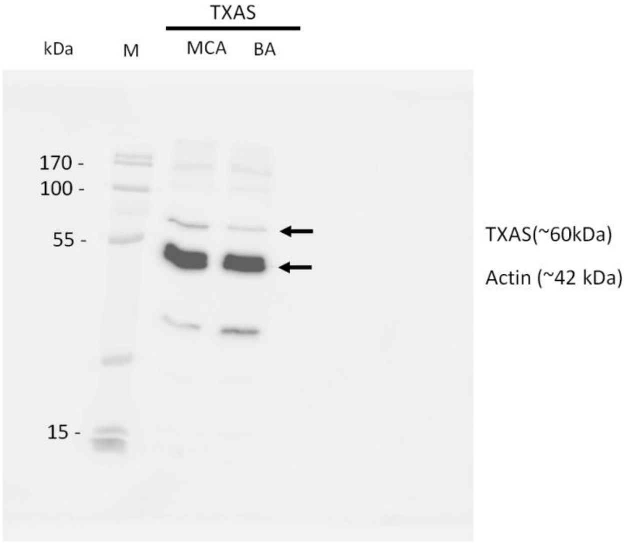

Expression of TXAS protein in rat middle cerebral (MCA) and basilar (BA) arteries

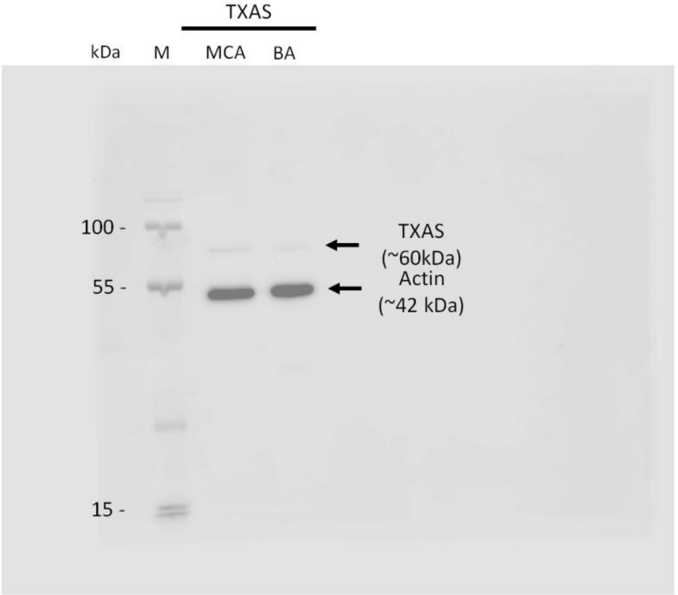

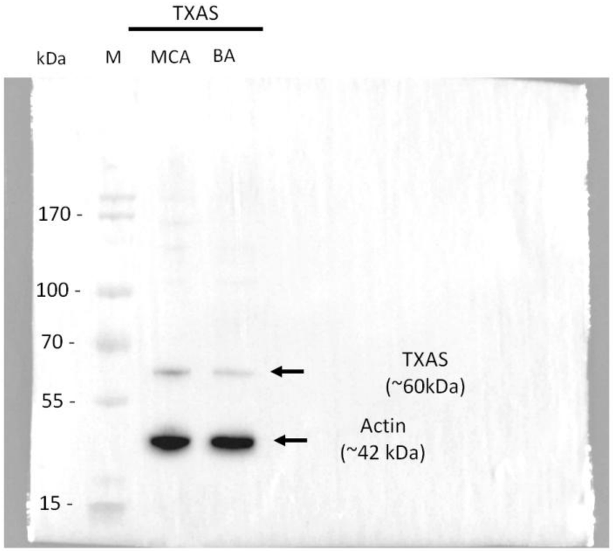

Expression of TXAS protein in rat middle cerebral (MCA) and basilar (BA) arteries

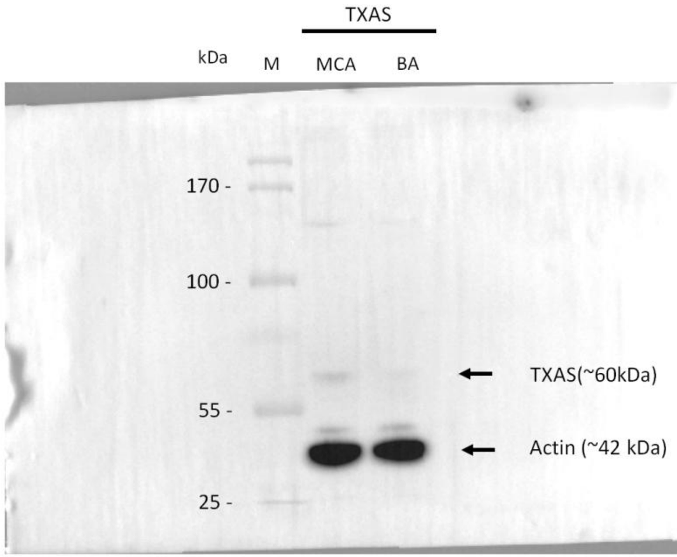

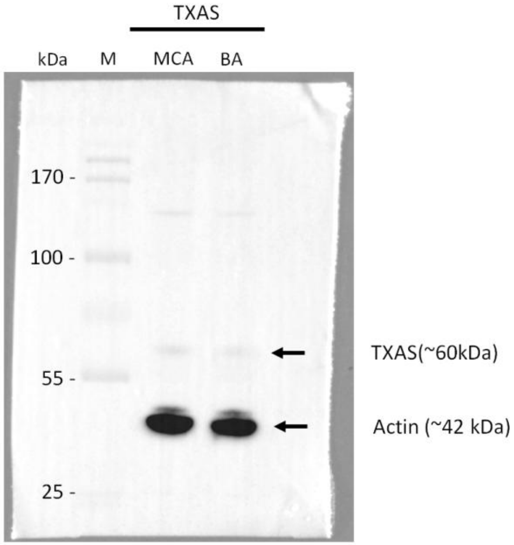

Expression of PGIS protein in rat middle cerebral (MCA) and basilar (BA) arteries

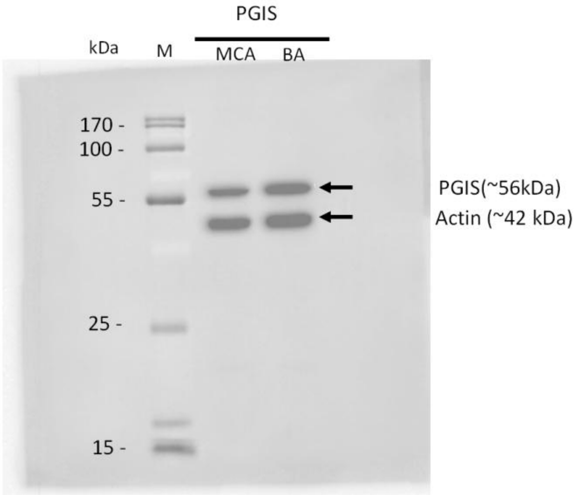

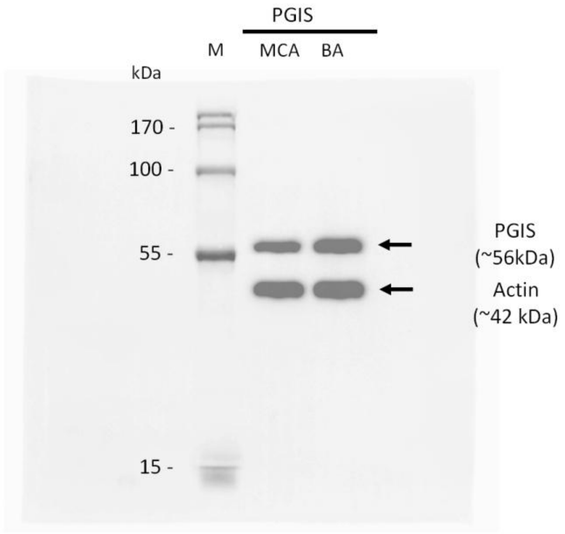

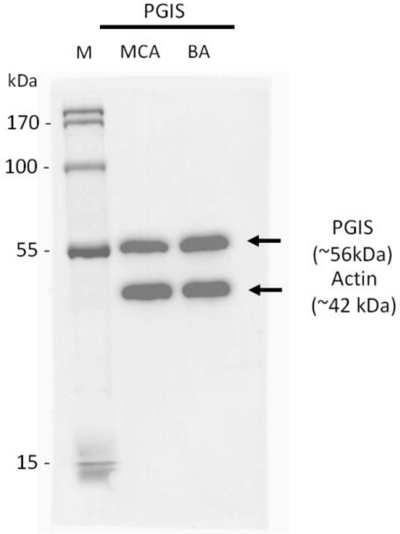

Expression of cytochrome P450 4A (Cyp450 4A) protein in rat middle cerebral (MCA) and basilar (BA) arteries

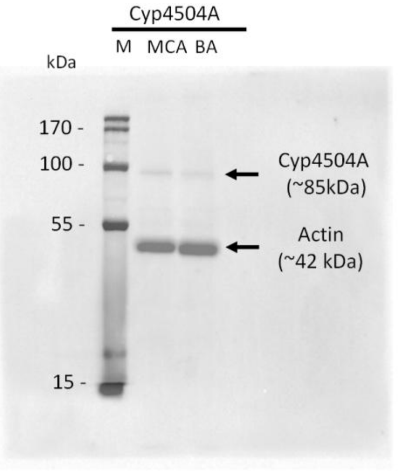

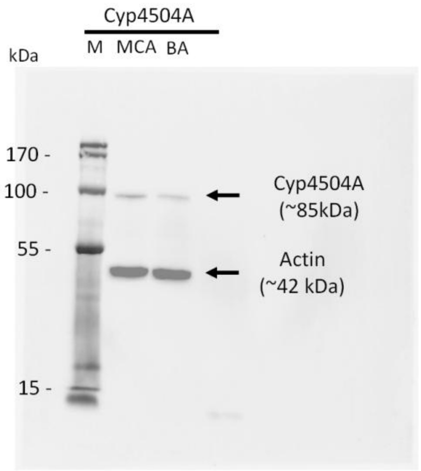

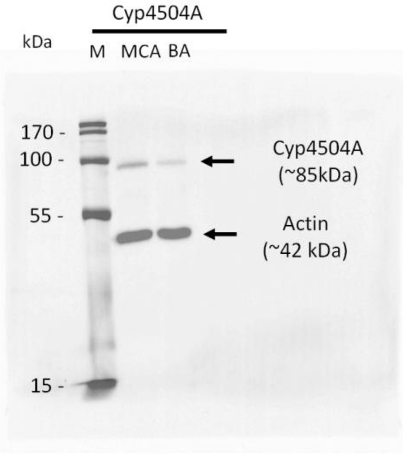

Expression of cytochrome prostacyclin receptor (PTGIR) protein in rat middle cerebral (MCA) and basilar (BA) arteries

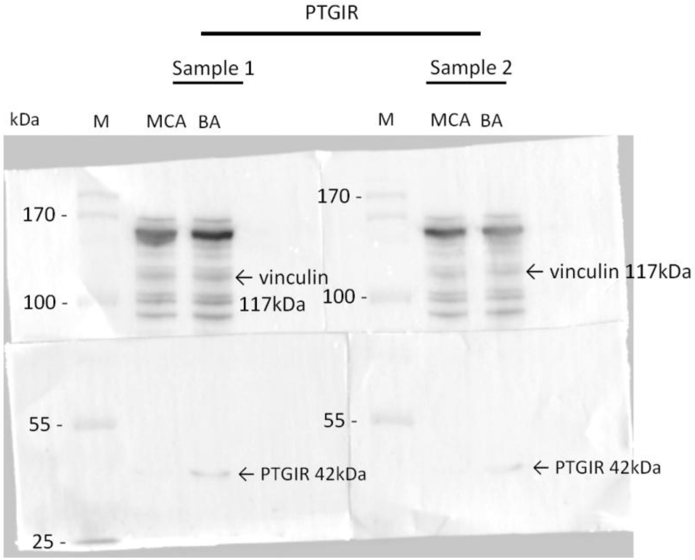

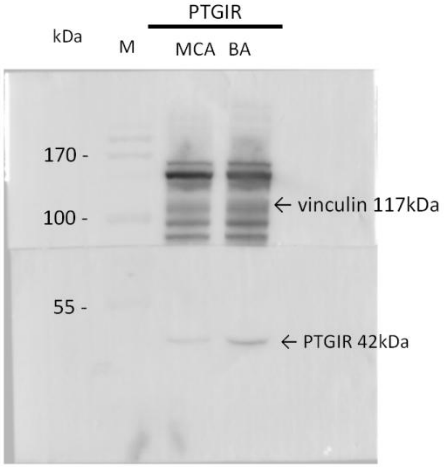

xpression of neuronal nitric oxide synthase (nNOS) protein in rat middle cerebral MCA) and basilar (BA) arteries

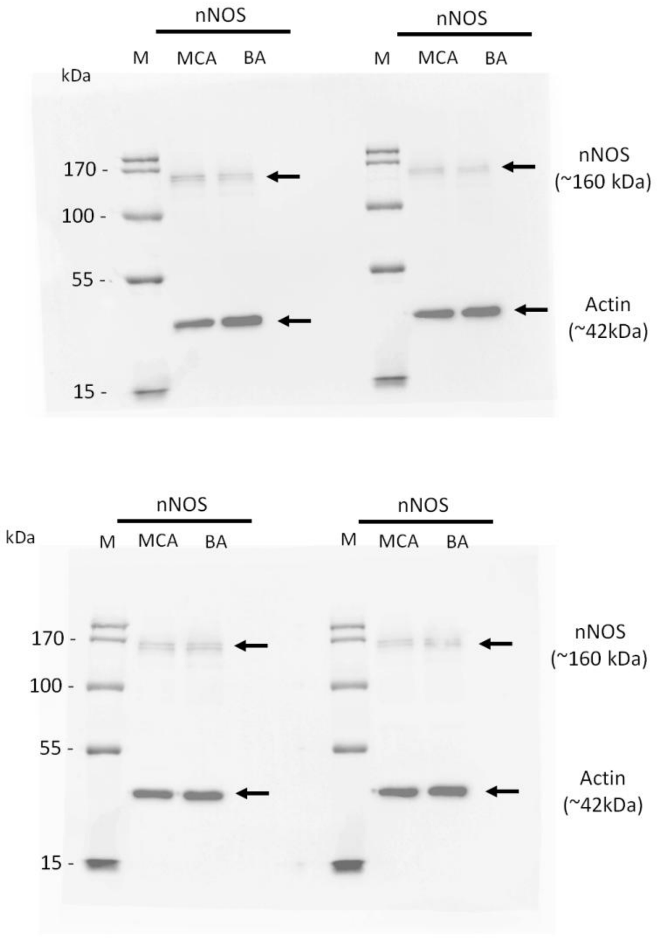

Expression of endothelial nitric oxide synthase (eNOS) protein in rat middle cerebral (MCA) and basilar (BA) arteries

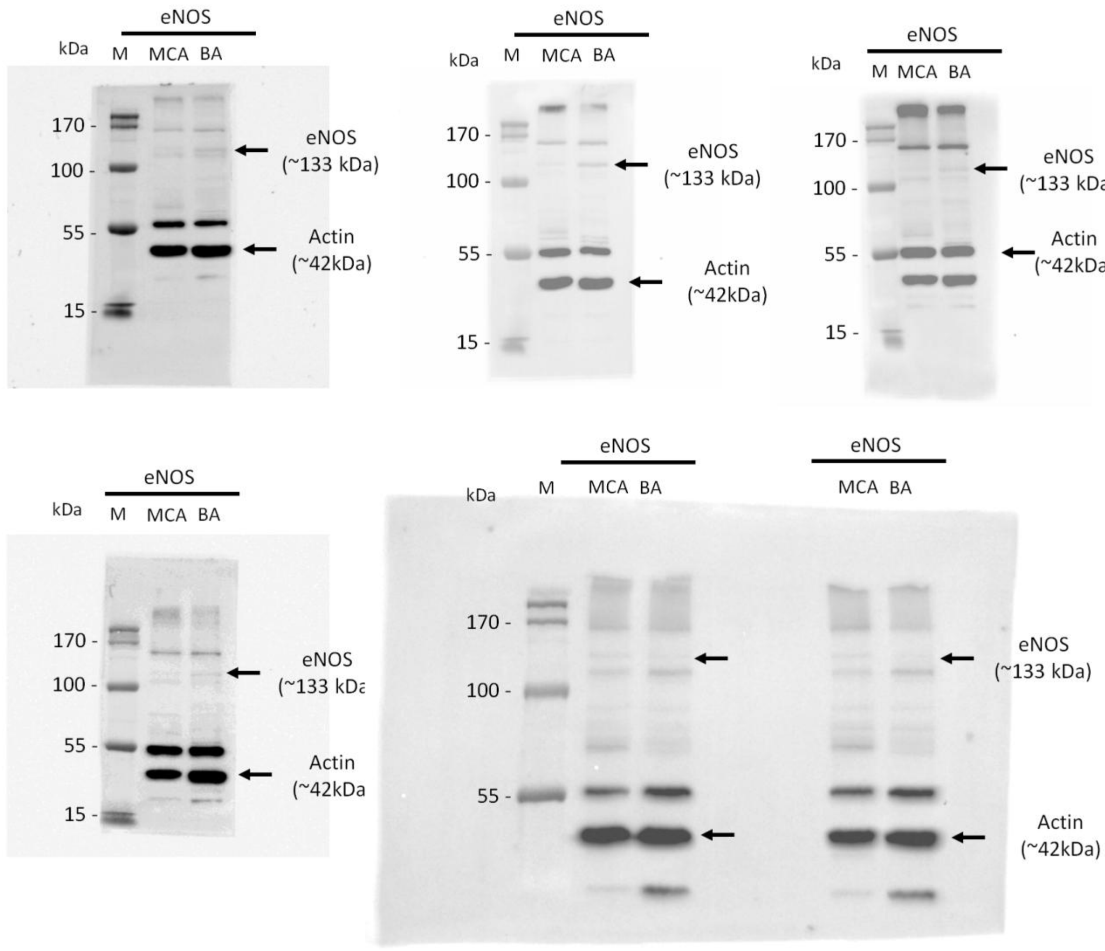

Supplement: Supplementary file 1 [file life-15-00856-s001.zip › Figure S1.pdf]
